# Identification and risk-factor analysis for individuals at high risk for keratoconus via machine learning and logistic regression

**DOI:** 10.1371/journal.pone.0354924

**Published:** 2026-08-25

**Authors:** Kaiyue Du, Rongmei Peng, Yueguo Chen, Bowei Yuan, Wei Chen, Liang Han, Gege Xiao, Yi Qu, Jing Hong

**Affiliations:** 1 Department of Ophthalmology, Peking University Third Hospital, Beijing, China; 2 Key Laboratory of Vision Loss and Restoration, Ministry of Education, Beijing, China; 3 Institute of Automation, Chinese Academy of Sciences, Beijing, China; University of Illinois at Chicago, UNITED STATES OF AMERICA

## Abstract

**Purpose:**

To evaluate keratoconus (KC) risk factors and to develop a machine-learning (ML) model for KC and myopia classification.

**Methods:**

In this retrospective single-center cross-sectional study, demographic and lifestyle data from patients with KC and individuals from a preoperative refractive surgery clinic were collected from January 20, 2024, to December 1, 2024. Univariable and multivariable regression analyses were used to identify key risk factors. Additionally, random forest (RF)-recursive feature elimination (RFE), extreme gradient boosting (XGBoost)-RFE, and univariable logistic regression were applied to select factors for ML models. Seven ML models were developed for a lifestyle-based classification system, with the performance being validated through discrimination and calibration, and interpretability being improved using SHapley Additive exPlanations (SHAP).

**Results:**

Analysis of 711 patients (mean [standard deviation] age, 26.6 [7.1] years; 439 males [61.7%]) revealed 275 with KC. Multivariable regression analysis identified seven risk factors for KC, including male sex, higher body-mass index (BMI), lower education level, more distant childhood residence, allergic conjunctivitis, and increased eye-rubbing intensity and frequency. After feature selection of 24 variables, the neural-network model demonstrated the highest performance (area under the receiver operating characteristic curve [AUROC] = 0.79), followed by RF (AUROC = 0.77) and XGBoost (AUROC = 0.76). SHAP analysis consistently highlighted eye-rubbing intensity, sex, BMI, and childhood residence among the top 10 factors across the top three models, which were also confirmed by univariable logistic regression.

**Conclusion:**

ML models can distinguish high-risk KC groups based on clinical risk factors, facilitating risk stratification and early lifestyle interventions.

## Introduction

Keratoconus (KC) is a degenerative corneal ectasia that is characterized by central or paracentral corneal protuberance and stromal thinning. KC causes progressive myopia, irregular astigmatism, and eventually critical visual impairment [1]. The annual prevalence and incidence rates of KC are estimated to be 0.2–4,790 per 100,000 people and 1.5–25 cases per 100,000 people, respectively, with the highest prevalence and incidence in people aged 20–30 years and in those of Middle Eastern and Asian descent [2]. The milder form of KC can be managed with vision aids that include spectacles and contact lenses; however, if these aids fail to improve vision, corneal surgery may be necessary, and treatments include corneal cross-linking, refractive surgery, corneal transplantation, or a combination of several refractive surgery procedures [2]. KC imposes a significant psychosocial and economic burden globally [3].

The pathological mechanism of KC has not been fully elucidated; however, it is generally understood to be multifactorial and influenced by a combination of environmental, genetic, auto-metabolic, viral infection, lifestyle, and immune factors [4]. Some studies have indicated that eye rubbing [5], atopy [6], body mass index (BMI) [7], sleeping position [8], exposure to ultraviolet (UV) light and air pollution [9], and a family history of the disease [10] are risk factors for KC; however, the influence of environmental factors on the onset of KC remains controversial.

Previous research on risk factors that are associated with KC predominantly relied on logistic regression, which provides easily interpretable results, thus aiding in the comprehension of every predictor’s impact on the outcome, albeit potentially struggling to capture intricate variable interactions [11]. In contrast, machine-learning (ML) techniques excel at analyzing large datasets by uncovering complex patterns, often yielding more accurate predictions than traditional statistical approaches [12]. Nevertheless, the “black box” nature of some ML models poses challenges for explanation [13]. To address this, we conducted a retrospective analysis of the demographics and lifestyle habits of patients with KC by utilizing a hybrid approach of logistic regression and ML. This allowed us to leverage the strengths of both methods, enhancing discrimination while maintaining model interpretability [14].

Our aim was to characterize a range of environmental, behavioral, and socioeconomic factors that may influence the onset of KC and to develop an explainable and trustworthy model for early risk stratification. Identifying and managing modifiable risk factors associated with KC could help reduce its incidence and alleviate the economic burden on society [5,15].

## Materials and methods

This retrospective study focused on patients with KC and myopia treated at the Centre for Sight of Peking University Third Hospital in Beijing, China, from January 20, 2024, to December 1, 2024. The study received approval from the Institutional Review Board of Peking University Third Hospital (No. M2023859) and adhered to the principles outlined in the Declaration of Helsinki. Written informed consent was obtained from every patient or the legal guardian of subjects who were under the age of 18 years.

### Inclusion criteria

Clinical inclusion criteria for KC were as follows: presence of at least one positive sign on slit-lamp examination (Munson’s sign, corneal scar, Vogt’s striae, or Fleischer’s ring), and an asymmetric bowtie pattern with or without skewed axes revealed by corneal tomography mapping [1].

The control group comprised individuals presenting to the preoperative refractive surgery clinic. All refractive surgery candidates included in this study were myopic (<−6.0 diopters), with or without astigmatism. Candidates underwent clinical and tomographical evaluation, and they were determined to be free of any signs of KC by the ophthalmologists involved in this study. Individuals with concurrent ocular conditions that include corneal ulcers, keratitis, cataracts, fundus lesions, trauma, and/or severe systemic diseases were not eligible for enrollment.

### Clinical data

All of the patients received preoperative visual acuity examination, slit lamp examination, fundoscopy, intraocular pressure measurement, autorefraction (Topcon RM 8800, Topcon Corporation, Tokyo, Japan), phoropter (Topcon CV-5000, Topcon Corporation, Tokyo, Japan), trial lenses, anterior segment optical coherence tomography (*Visante*, Zeiss, Oberkochen, Germany), corneal tomography (Pentacam, Oculus, Washington, DC, USA), and biomechanical measurements utilizing the Corvis ST (Oculus; Optikgeräte GmbH, Wetzlar, Germany).

Height and weight were measured with participants wearing light clothing and no shoes via a wall-mounted stadiometer and an electronic scale, respectively. BMI was calculated from the height and weight values using the standard formula (weight [kg]/height [m]^2^).

### Questionnaire survey

The survey was conducted by trained staff in face-to-face interviews with patients at the clinic during the initial visit to the hospital for the study. The demographic questionnaire collected information on age, sex, place of birth, dominant hand, education level, occupational status, childhood residence, childhood family socioeconomic status, history of eye rubbing, sleeping position, snoring and sleep apnea, exposure to dust or UV light, screen time and reading habits, life stress, smoking and alcohol consumption, allergy and allergic disease history, family history of KC, pregnancy history and hormone medication usage, as well as history of eye and systemic diseases. For patients with KC, information on the age of onset and diagnosis, symptoms, and management strategies was also recorded. The severity of KC was classified into three levels based on the treatment approach, namely, mild, moderate, and severe. Mild cases were managed with glasses or contact lenses, moderate cases underwent corneal collagen cross-linking, and severe cases required corneal transplantation.

### Statistical analysis

**Fig 1:**
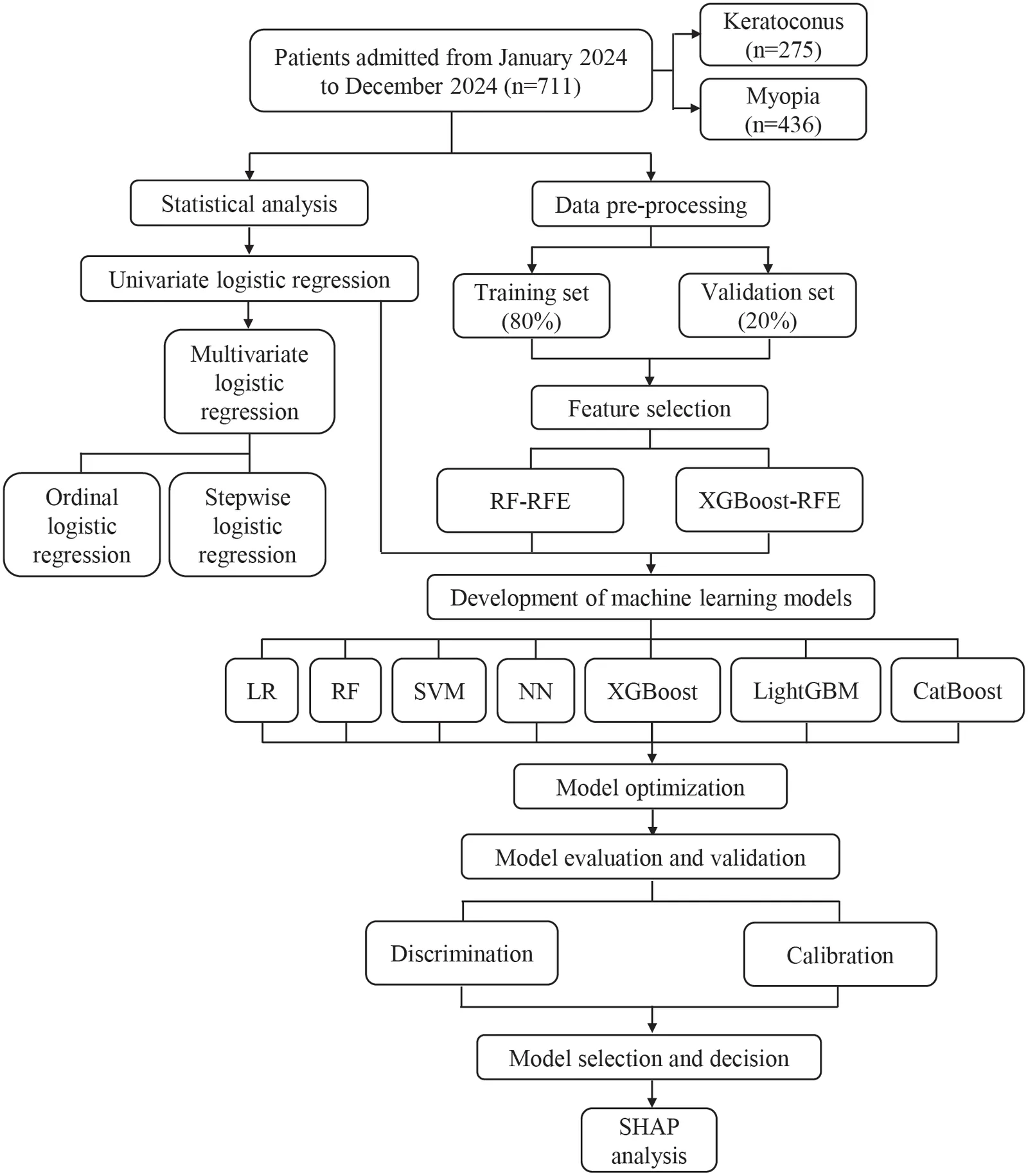
Study flowchart. CatBoost, categorical boosting; LightGBM, light gradient-boosting machine; LR, logistic regression; NN, neural network; RF, random forest; RFE, recursive feature elimination; SHAP, SHapley Additive exPlanations; SVM, support vector machines; XGBoost, extreme gradient boosting.

We used the SPSS 25.0 software package (SPSS Inc, Chicago, USA) for data analysis. Descriptive statistics are reported as mean (standard deviation) for continuous variables and as number (%) for categorical variables. The normality of all data samples was assessed using the Shapiro-Wilk test. An independent Student’s t-test was utilized for age, while the exact Wilcoxon rank-sum test was applied to other continuous variables. The Chi-squared test was employed for comparisons of categorical data. A two-tailed *P* value of less than 0.05 was considered statistically significant. The overall flowchart of the research is shown in Fig 1.

### Logistic regression

Single-factor logistic regression analysis was first conducted to identify potential risk factors. After that, significant variables were incorporated into multiple-factor logistic regression and stepwise logistic regression analyses. The disease severity variable was categorized into four ordered groups, namely, “normal,” “mild,” “moderate,” and “severe,” for ordered multinomial logistic regression, in order to investigate the risk factors related to KC severity.

Further, we explored the interaction effects between eye rubbing and allergic conjunctivitis (AC), as well as eye rubbing and BMI, on the multiplicative scale by introducing interaction terms into the stepwise logistic regression model. In addition, additive interaction was evaluated by calculating the relative excess risk from the interaction according to the estimated effect measures. The classification performance of the regression model was evaluated utilizing the receiver operating characteristic curve (ROC).

### ML

The data preprocessing involved standardizing continuous variables, ordinal encoding for ordinal categorical variables, and one-hot encoding for nominal categorical variables. There were no missing data in the dataset, which were randomly split at the subject level into training and test sets at an 80:20 ratio, respectively.

To identify key factors, we applied random forest (RF)-recursive feature elimination (RFE), extreme gradient boosting (XGBoost)-RFE, and univariable logistic regression analysis exclusively on the training set, selecting factors that appeared in at least two out of the three screening methods [16]. The Synthetic Minority Oversampling Technique was utilized only on the training set to address class imbalance [17].

Seven ML models were employed: logistic regression (LR), RF, support vector machine (SVM), XGBoost, categorical boosting (CatBoost), light gradient boosting machine (LightGBM), and neural networks (NN). Hyperparameter tuning for all models was performed using Bayesian optimization with fivefold cross-validation within the training set.

The feedforward neural network model comprises three linear layers with two Parametric Rectified Linear Unit activation functions. It is designed as a four-layer deep learning model (24–128–128–1) for binary output, consisting of an input layer with 24 inputs and two hidden layers with 128 and 128 nodes. Dropout regularization is implemented after every activation function to mitigate overfitting. The model was implemented using PyTorch and trained utilizing the AdamW optimizer for 500 epochs, with a learning rate of 0.001. The loss function employed was BCEWithLogitsLoss.

The ML models produced both deterministic and probabilistic outputs. The models’ accuracy, sensitivity, specificity, F1 score, area under the receiver operating characteristic curve (AUROC), and area under the precision-recall curve (AUPRC) were determined to assess their discriminative power. A classification threshold of 0.5 was used for all models. Accuracy, sensitivity, and specificity were calculated with 95% confidence intervals (CI) determined using the Wilson method [18]. CIs for the F1 score, AUROC, AUPRC, and Brier score were estimated using bootstrapping. An AUROC value of 0.50–0.60 was considered poor, 0.60–0.70 was subpar, 0.70–0.80 was fair, and 0.80–0.90 was good [19]. Calibration was assessed using the Brier score. Pairwise AUROC comparisons were conducted using the DeLong test, with significance set at *p* < 0.05.

Model interpretability was further enhanced through SHapley Additive exPlanations (SHAP) [20], which were calculated for the top three models to illustrate the contribution of each feature to the model’s classification.

## Results

### Baseline characteristics

The analysis of 711 patients (mean [standard deviation] age, 26.6 [7.1] years; 439 males [61.7%]) identified 275 (38.7%) with KC. Demographic data and eye examination results for both groups are presented in Table 1. For KC patients diagnosed in one eye, data from the affected eye were used. For those diagnosed in both eyes and control subjects, data from one randomly selected eye were analyzed.

**Table 1:** Demographic data and eye examination results for the keratoconus and control groups.

Characteristic	Individuals^a^		*p* value^b^
	KC (n = 275)	Control (n = 436)	
**Sex**			
Female	71 (25.8)	201 (46.1)	< 0.001
Male	204 (74.2)	235 (53.9)	< 0.001
**Age, years**	26.7 (7.2)	26.6 (7.1)	0.85
**SE, D**	−6.64 (4.20)	−4.41 (2.07)	< 0.001
**CYL, D**	−4.13 (4.06)	−0.92 (0.83)	< 0.001
**CDVA, logMAR**	0.41 (0.38)	0.01 (0.02)	< 0.001
**IOP, mmHg**	12.62 (2.81)	16.68 (2.25)	< 0.001
**Kmean, D**	51.65 (9.77)	43.22 (1.45)	< 0.001
**Kmax, D**	61.90 (14.10)	44.58 (1.72)	< 0.001
**TCT, μm**	442.6 (61.8)	543.6 (28.6)	< 0.001
**CCT, μm**	480.7 (144.8)	558.3 (28.5)	< 0.001
**CBI**	0.89 (0.26)	0.02 (0.10)	< 0.001

Abbreviations: KC, keratoconus; SE, spherical equivalent; CYL, refractive cylinder; CDVA, corrected distance visual acuity; IOP, intraocular pressure; Kmean, mean keratometry; Kmax, max keratometry; TCT, thinnest corneal thickness measured with Pentacam; CCT, central corneal thickness measured with anterior segment optical coherence tomography; CBI, Corvis biomechanical index.

^a^Sex distribution is reported as the number (percentage) for each group, whereas the mean (standard deviation) of other variables is provided.

^b^Age was compared using Student’s *t* test, sex was compared using the Chi-squared test, and other variables were compared using the exact Wilcoxon rank-sum test.

### Univariable logistic regression

In univariable analysis, 32 variables were examined, revealing 12 significant risk factors for KC, namely, sex, BMI, education level, childhood residence, childhood family socioeconomic status, eye rubbing frequency, duration, intensity, use of eye coverings during sleep, snoring and sleep apnea, AC, and work or study stress. In addition, specific investigations within the female group indicated that hormone medication usage and pregnancy history were not correlated with KC occurrence (Table 2).

**Table 2:** Univariable analysis of factors related to keratoconus.

Categories	Characteristics	No. (%)		*p* value^a^	OR (95% CI)
		Keratoconus (n = 275)	Control (n = 436)		
**Sex**	Male	204 (74.2)	235 (53.9)	< 0.001	2.46 (1.77–3.42)
	Female	71 (25.8)	201 (46.1)	Reference	
**Age, years**	< 18	23 (8.4)	40 (9.2)	0.97	1.01 (0.50–2.06)
	18–35	227 (82.5)	352 (80.7)	0.63	1.14 (0.68–1.91)
	> 35	25 (9.1)	44 (10.1)	Reference	
**BMI, kg/m** ^**2**^	< 18.5	18 (6.5)	47 (10.8)	< 0.001	0.20 (0.09–0.43)
	18.5–23.9	145 (52.7)	276 (63.3)	< 0.001	0.27 (0.15–0.49)
	24.0–27.9	75 (27.3)	94 (21.6)	0.01	0.41 (0.22–0.77)
	≥ 28	37 (13.5)	19 (4.4)	Reference	
**Dominant hand**	Left	20 (7.3)	29 (6.7)	0.75	1.10 (0.61–1.99)
	Right	255 (92.7)	407 (93.3)	Reference	
**Education level**	High school or below	42 (15.3)	26 (6.0)	< 0.001	2.84 (1.61–5.02)
	Undergraduate	163 (59.3)	287 (65.8)	0.99	1.00 (0.70–1.42)
	Master’s degree or above	70 (25.5)	123 (28.2)	Reference	
**Occupational status**	Unemployed/Retired	13 (4.7)	11 (2.5)	0.15	1.85 (0.80–4.33)
	Student	93 (33.8)	183 (42.0)	0.18	0.79 (0.57–1.11)
	Non-office work	43 (15.6)	45 (10.3)	0.10	1.49 (0.93–2.40)
	Office work	126 (45.8)	197 (45.2)	Reference	
**Childhood residence**	Rural	109 (39.6)	102 (23.4)	< 0.001	2.32 (1.20–3.58)
	Small city	66 (24.0)	107 (24.5)	0.22	1.34 (0.84–2.12)
	Medium city	29 (10.5)	61 (14.0)	0.92	1.03 (0.59–1.80)
	Large city	23 (8.4)	62 (14.2)	0.47	0.80 (0.45–1.45)
	Megacity	48 (17.5)	104 (23.9)	Reference	
**Childhood family socioeconomic status**	Poor	44 (16.0)	35 (8.0)	< 0.001	2.72 (1.59–4.65)
	Average	169 (61.5)	267 (61.2)	0.09	1.37 (0.96–1.96)
	Good/Excellent	62 (22.5)	134 (30.7)	Reference	
**Family history of keratoconus**	Yes	6 (2.2)	3 (0.7)	0.10	3.22 (0.80–12.98)
	No	269 (97.8)	433 (99.3)	Reference	
**Eye rubbing frequency**	Never/Rarely	51 (18.5)	104 (23.9)	< 0.001	0.25 (0.15–0.43)
	Occasionally	74 (26.9)	173 (39.7)	< 0.001	0.22 (0.13–0.36)
	Sometimes	84 (30.5)	125 (28.7)	< 0.001	0.35 (0.21–0.57)
	Frequently/Always	66 (24.0)	34 (7.8)	Reference	
**Eye rubbing duration**	<10 seconds	226 (82.2)	405 (92.9)	< 0.001	0.35 (0.22–0.57)
	≥10 seconds	49 (17.8)	31 (7.1)	Reference	
**Eye rubbing intensity**	Light	104 (37.8)	288 (66.1)	< 0.001	0.12 (0.06–0.23)
	Medium	132 (48.0)	135 (31.0)	0.001	0.33 (0.17–0.64)
	Heavy	39 (14.2)	13 (3.0)	Reference	
**Rubbed eye area**	Eye corner	76 (27.6)	146 (33.5)	0.19	0.78 (0.54–1.13)
	Eyelid	87 (31.6)	122 (28.0)	0.72	1.07 (0.74–1.54)
	No difference	112 (40.7)	168 (38.5)	Reference	
**Eye rubbing position**	Fingertip	78 (28.4)	126 (28.9)	0.11	0.55 (0.27–1.14)
	Knuckle	142 (51.6)	242 (55.5)	0.07	0.52 (0.26–1.06)
	Whole fingertip	37 (13.5)	52 (11.9)	0.26	0.63 (0.29–1.40)
	Palm root/Whole fist	18 (6.5)	16 (3.7)	Reference	
**Sleeping position**	Back position	83 (30.2)	134 (30.7)	0.74	0.90 (0.46–1.73)
	Side position	174 (63.3)	276 (63.3)	0.77	0.91 (0.49–1.71)
	Prone position	18 (6.5)	26 (6.0)	Reference	
**Use of eye coverings during sleep**	Yes	35 (12.7)	31 (7.1)	0.01	1.91 (1.15–3.17)
	No	240 (87.3)	405 (92.9)	Reference	
**Snoring and sleep apnea**	Yes	68 (24.7)	73 (16.7)	0.01	1.63 (1.13–2.37)
	No	207 (75.3)	363 (83.3)	Reference	
**Daily electronic screen time, hours**	0	6 (2.2)	4 (0.9)	0.22	2.29 (0.62–8.53)
	1–3	33 (12)	39 (8.9)	0.39	1.29 (0.72–2.32)
	4–6	101 (36.7)	163 (37.4)	0.81	0.95 (0.62–1.46)
	7–9	84 (30.5)	152 (34.9)	0.46	0.85 (0.54–1.32)
	≥10	51 (18.5)	78 (17.9)	Reference	
**Dark environment screen time, hours**	0	77 (28.0)	104 (23.9)	0.13	3.33 (0.70–15.86)
	1–2	166 (60.4)	269 (61.7)	0.20	2.78 (0.59–13.01)
	3–4	25 (9.1)	46 (10.6)	0.28	2.45 (0.49–12.21)
	5–6	5 (1.8)	8 (1.8)	0.28	2.81 (0.42–18.74)
	≥7	2 (0.7)	9 (2.1)	Reference	
**Daily paper material reading time, hours**	0	75 (27.3)	105 (24.1)	0.93	0.95 (0.32–2.86)
	1–3	158 (57.5)	276 (63.3)	0.62	0.76 (0.26–2.24)
	4–6	27 (9.8)	39 (8.9)	0.89	0.92 (0.29–2.96)
	7–9	9 (3.3)	8 (1.8)	0.58	1.50 (0.36–6.23)
	≥10	6 (2.2)	8 (1.8)	Reference	
**Daily sunlight/UV exposure time, hours**	0	32 (11.6)	44 (10.1)	0.68	1.46 (0.25–8.43)
	1–3	215 (78.2)	356 (81.7)	0.83	1.21 (0.22–6.65)
	4–6	22 (8.0)	24 (5.5)	0.51	1.83 (0.31–11.02)
	7–9	4 (1.5)	8 (1.8)	> 0.99	1.00 (0.13–8.00)
	≥10	2 (0.7)	4 (0.9)	Reference	
**Nighttime work/study**	Yes	109 (39.6)	183 (42.0)	0.54	0.91 (0.67–1.24)
	No	166 (60.4)	253 (58.0)	Reference	
**Work/study stress**	Yes	156 (56.7)	204 (46.8)	0.01	1.49 (1.10–2.02)
	No	119 (43.3)	232 (53.2)	Reference	
**Exposure to dust**	Yes	8 (2.9)	7 (1.6)	0.25	1.84 (0.66–5.12)
	No	267 (97.1)	429 (98.4)	Reference	
**Smoking**	Yes	34 (12.4)	52 (11.9)	0.86	1.04 (0.66–1.65)
	No	241 (87.6)	384 (88.1)	Reference	
**Alcohol consumption**	Yes	78 (28.4)	131 (30.0)	0.63	0.92 (0.66–1.29)
	No	197 (71.6)	305 (70.0)	Reference	
**Allergic conjunctivitis**	Yes	23 (8.4)	19 (4.4)	0.03	2.00 (1.07–3.75)
	No	252 (91.6)	417 (95.6)	Reference	
**Allergic diseases**	Yes	94 (34.2)	141 (32.3)	0.61	1.09 (0.79–1.50)
	No	181 (65.8)	295 (67.7)	Reference	
**Allergies to substances**	Yes	66 (24.0)	102 (23.4)	0.85	1.03 (0.73–1.47)
	No	209 (76.0)	334 (76.6)	Reference	
**Thyroid disease**	Yes	7 (2.5)	14 (3.2)	0.61	0.79 (0.31–1.98)
	No	268 (97.5)	422 (96.8)	Reference	
**Pregnancy history**	Yes	24 (33.8)	71 (35.3)	0.86	0.95 (0.51–1.74)
	No	47 (66.2)	130 (64.7)	Reference	
**Hormone medication usage**	Yes	9 (12.7)	34 (16.9)	0.45	0.72 (0.32–1.66)
	No	62 (87.3)	167 (83.1)	Reference	

Abbreviations: BMI, body mass index; CI, confidence interval; OR, odds ratio; UV, ultraviolet.

^a^Comparisons were made through univariable analysis.

### Multivariable logistic regression

The 12 previously identified factors underwent multiple-factor logistic regression analysis. The results indicated that sex, BMI, education level, childhood residence, eye rubbing frequency and intensity, as well as a history of AC, are independent risk factors for KC (Table 3). Additionally, incorporating these variables into the stepwise logistic regression confirmed the same seven factors identified in the standard logistic regression, reinforcing the reliability of the findings. Ordinal logistic regression was then employed to investigate factors influencing KC severity. The interactive effects of eye rubbing and AC history, as well as the interaction between eye rubbing and BMI, were also analyzed. Further details are provided in the S1 File.

**Table 3:** Multivariable logistic regression analysis of risk factors for keratoconus.

Categories	Characteristics	b	SE (b)	*p* value^a^	OR (95% CI)
**Sex**	Male	0.86	0.20	< 0.001	2.37 (1.60–3.52)
	Female	Reference			
**BMI, kg/m** ^**2**^	<18.5	−1.08	0.46	0.02	0.34 (0.14–0.84)
	18.5–23.9	−0.96	0.34	0.01	0.39 (0.20–0.75)
	24.0–27.9	−0.67	0.36	0.07	0.51 (0.25–1.04)
	≥28	Reference			
**Education level**	High school or below	0.69	0.34	0.04	1.99 (1.03–3.88)
	Undergraduate	–0.19	0.21	0.36	0.83 (0.55–1.24)
	Master’s degree or above	Reference			
**Childhood residence**	Rural	0.99	0.28	< 0.001	2.68 (1.54–4.65)
	Small city	0.51	0.29	0.08	1.66 (0.95–2.90)
	Medium city	0.17	0.33	0.60	1.19 (0.62–2.25)
	Large city	−0.09	0.34	0.80	0.92 (0.47–1.79)
	Megacity	Reference			
**Childhood family socioeconomic status**	Poor	0.20	0.34	0.55	1.22 (0.63–2.38)
	Average	0.06	0.23	0.80	1.06 (0.68–1.65)
	Good/Excellent	Reference			
**Eye rubbing frequency**	Never/Rarely	−0.41	0.34	0.22	0.66 (0.34–1.28)
	Occasionally	−0.72	0.31	0.02	0.49 (0.27–0.89)
	Sometimes	−0.50	0.30	0.09	0.61 (0.34–1.09)
	Frequently/Always	Reference			
**Eye rubbing duration**	<10 seconds	−0.42	0.30	0.16	0.66 (0.37–1.17)
	≥10 seconds	Reference			
**Eye rubbing intensity**	Light	−1.70	0.40	< 0.001	0.18 (0.08–0.40)
	Medium	−0.91	0.39	0.02	0.40 (0.19–0.86)
	Heavy	Reference			
**Use of eye coverings during sleep**	Yes	0.33	0.30	0.27	1.40 (0.77–2.53)
	No	Reference			
**Snoring and sleep apnea**	Yes	−0.06	0.23	0.79	0.94 (0.60–1.48)
	No	Reference			
**Work/Study stress**	Yes	0.26	0.18	0.16	1.29 (0.90–1.85)
	No	Reference			
**Allergic conjunctivitis**	Yes	0.80	0.38	0.04	2.23 (1.06–4.71)
	No	Reference			

Abbreviations: BMI, body mass index; CI, confidence interval; OR, odds ratio; SE, standard error.

^a^Comparisons were made by multivariable logistic regression.

### Evaluation of the statistical logistic regression model

**Fig 2:**
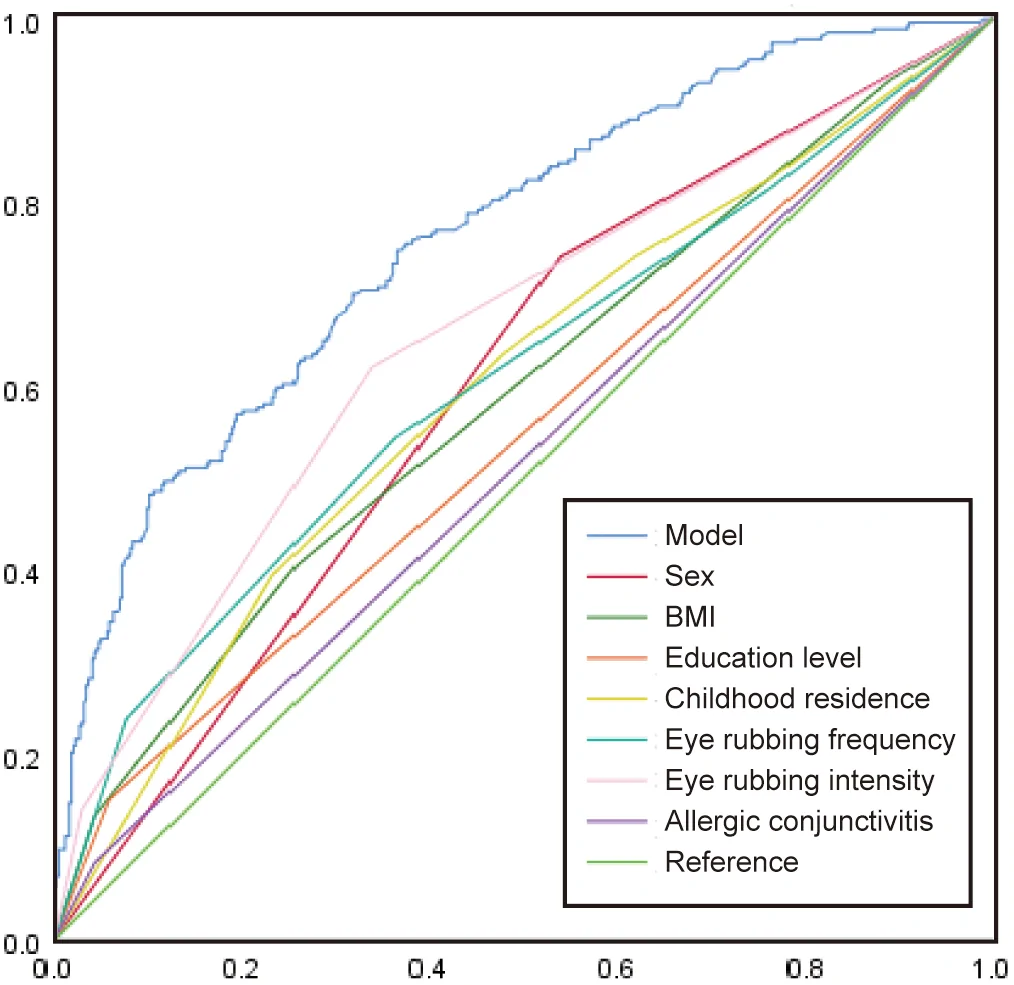
Receiver operating characteristic curves for the multivariable logistic regression analysis of seven risk factors and the joint model. BMI, body mass index.

The AUROC was 0.763 (95% CI, 0.727–0.798). Of the seven indicators, BMI, eye rubbing frequency, and intensity showed the highest diagnostic efficacy, as demonstrated by the single-factor AUROC for diagnosis. Fig 2 presents an overview of the ROC curves for the seven metrics and the combined model.

### Feature selection

**Fig 3:**
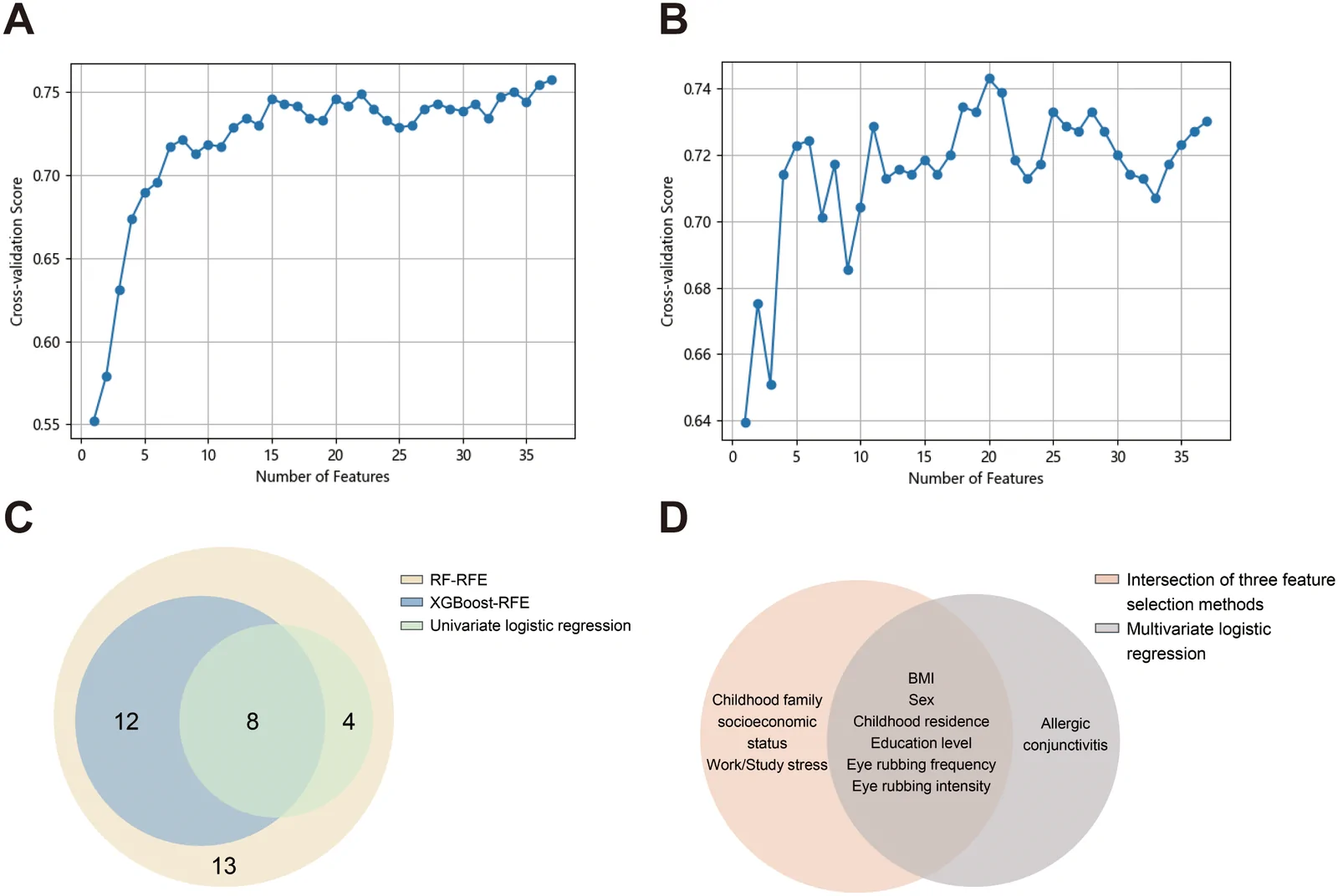
Screening of characteristic variables. **(A)** Feature selection using RF-RFE. **(B)** Feature selection using XGBoost-RFE. **(C)** Venn diagram comparing features selected by RF-RFE, XGBoost-RFE, and univariable logistic regression. **(D)** Venn diagram comparing the intersection of features selected by RF-RFE, XGBoost-RFE, and univariable logistic regression with those identified by multivariable logistic regression. BMI, body mass index; RF, random forest; RFE, recursive feature elimination; XGBoost, extreme gradient boosting.

A total of 37 feature variables, derived from the initial 32 encoded variables, were evaluated. The RF classifier retained all 37 features, while 20 features were selected for the XGBoost classifier using the RFE algorithm (Fig 3A and 3B). The union of features selected by XGBoost-RFE and univariable logistic regression was included in subsequent ML analyses (Fig 3C). The intersection of features identified by these three methods reveals that six out of eight overlap with those selected by multivariable logistic regression (Fig 3D).

### Evaluation of the ML model

**Fig 4:**
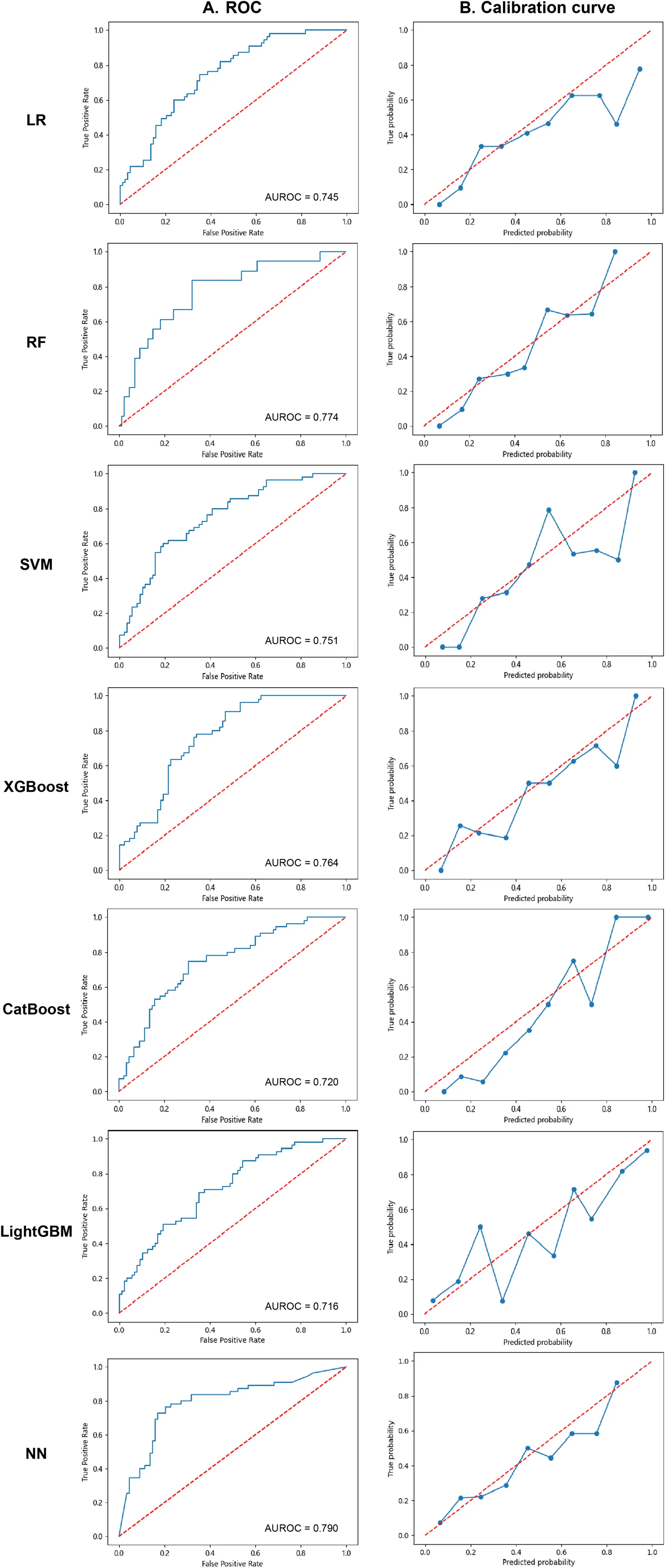
Performance evaluation of the machine learning models. **(A)** ROC curves of the seven models. **(B)** Calibration curves of the models, illustrating the agreement between predicted probabilities (x-axis) and observed probabilities (y-axis). The diagonal line represents perfect calibration. AUROC, area under the receiver operating characteristic curve; CatBoost, categorical boosting; LightGBM, light gradient boosting machine; LR, logistic regression; NN, neural network; RF, random forest; ROC, receiver operating characteristic curve; SVM, support vector machine; XGBoost, extreme gradient boosting.

A total of 24 variables were incorporated into the ML process (S4 Table), and seven models were used and evaluated for comparison (Table 4). NN demonstrated the highest discrimination performance (AUROC: 0.790; AUPRC: 0.697). The RF and XGBoost models followed, with AUROC values of 0.774 and 0.764, respectively (Fig 4). Furthermore, NN exhibited the strongest calibration, as indicated by a Brier score of 0.182. In addition, pairwise comparisons of AUROC using the DeLong test showed that the differences between models were not significant (all *p* > 0.05).

**Table 4:** Statistical comparisons among machine learning models.

Models	Accuracy (95% CI)	Sensitivity (95% CI)	Specificity (95% CI)	F_1_ score (95% CI)	AUROC (95% CI)	AUPRC (95% CI)	Brier score (95% CI)
**LR**	0.664 (0.584–0.737)	0.636 (0.504–0.751)	0.682 (0.579–0.770)	0.668 (0.591–0.733)	0.745 (0.672–0.807)	0.633 (0.552–0.708)	0.208 (0.186–0.234)
**RF**	0.748 (0.671–0.812)	0.546 (0.415–0.670)	0.875 (0.790–0.929)	0.739 (0.662–0.801)	0.774 (0.705–0.833)	0.664 (0.583–0.735)	0.186 (0.167–0.213)
**SVM**	0.741 (0.664–0.806)	0.600 (0.468–0.719)	0.830 (0.738–0.894)	0.737 (0.660–0.798)	0.751 (0.685–0.816)	0.652 (0.570–0.726)	0.194 (0.172–0.218)
**XGBoost**	0.713 (0.634–0.781)	0.655 (0.523–0.766)	0.750 (0.650–0.829)	0.715 (0.643–0.784)	0.764 (0.697–0.826)	0.698 (0.622–0.768)	0.191 (0.171–0.216)
**CatBoost**	0.699 (0.620–0.768)	0.564 (0.433–0.686)	0.784 (0.687–0.857)	0.696 (0.618–0.763)	0.720 (0.653–0.787)	0.683 (0.600–0.751)	0.203 (0.183–0.228)
**LightGBM**	0.671 (0.591–0.743)	0.509 (0.381–0.636)	0.773 (0.675–0.848)	0.666 (0.592–0.734)	0.716 (0.652–0.781)	0.601 (0.523–0.684)	0.230 (0.206–0.255)
**NN**	0.783 (0.692–0.839)	0.691 (0.506–0.792)	0.841 (0.731–0.901)	0.782 (0.701–0.845)	0.790 (0.719–0.850)	0.697 (0.609–0.771)	0.182 (0.159–0.205)

Abbreviations: AUPRC, area under the precision-recall curve; AUROC, area under the receiver operating characteristic curve; CatBoost, categorical boosting; CI, confidence interval; LightGBM, light gradient boosting machine; LR, logistic regression; NN, neural network; RF, random forest; SVM, support vector machine; XGBoost, extreme gradient boosting.

### SHAP analysis

**Fig 5:**
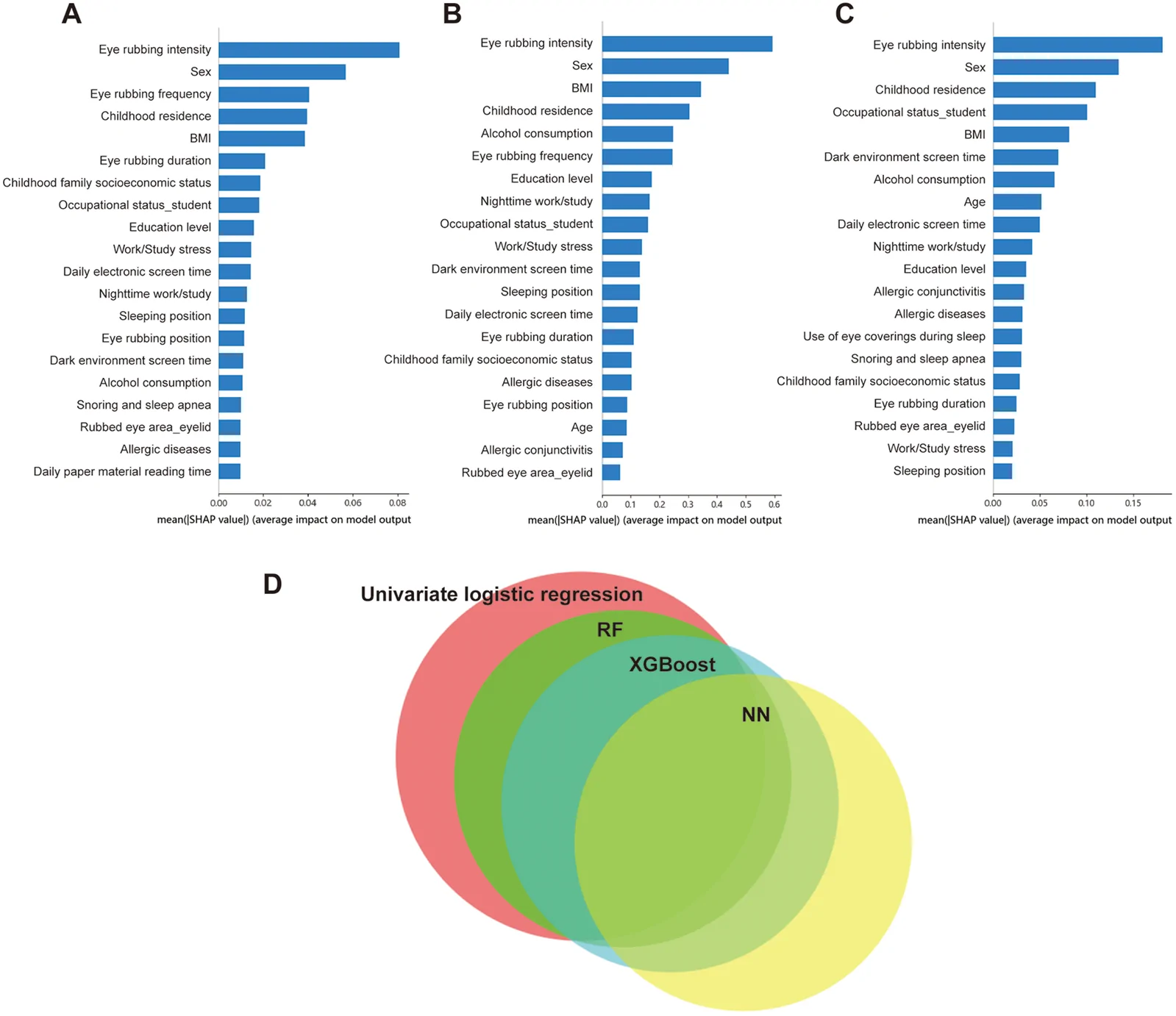
SHAP analysis of feature importance for the top three models. **(A)** SHAP values for the 20 most important features in the RF model. **(B)** SHAP values for the 20 most important features in the XGBoost model. **(C)** SHAP values for the 20 most important features in the NN model. **(D)** Venn diagram comparing the top 10 features from the three models and those identified by univariable logistic regression. BMI, body-mass index; NN, neural network; RF, random forest; SHAP, SHapley Additive exPlanation; XGBoost, extreme gradient boosting.

Fig 5 displays the feature importance for the top three models, highlighting the mean absolute SHAP values for the 20 most significant features, ranked in descending order. Eye-rubbing intensity ranks as the most important feature across all three algorithms. Additionally, sex, BMI, and childhood residence consistently appear among the top 10 features across all models and are also identified through univariable logistic regression. Of the 12 features identified by univariable logistic regression, 9, 7, and 4 overlap with the top 10 features in the RF, XGBoost, and NN models, respectively (Fig 5D).

## Discussion

This paper explored the risk factors of KC and established a theoretical foundation for clinical practitioners to perform early assessments of KC onset. Prior studies have used ML methods to study the onset of certain diseases linked to their causes and risk factors [21,22]; however, this study presents the initial known instance of integrating traditional logistic regression with ML techniques to analyze the risk factors related to KC.

This study used logistic regression to analyze 32 factors associated with the onset of KC, identifying sex, BMI, education level, childhood residence, eye rubbing frequency and intensity, and a history of AC as independent risk factors. Men exhibited twice the prevalence and more severe symptoms of KC than women did, although sex-based prevalence varied across different populations [7,23]. High BMI was associated with more severe KC, potentially owing to obesity-induced eyelid weakening [7], persistent ocular surface inflammation [24], and increased susceptibility to corneal damage. BMI may also serve as a surrogate marker for conditions such as metabolic syndrome or floppy eyelid syndrome, which have been reported to be associated with KC [25,26]. We observed significant multiplicative interactions between BMI and several eye-rubbing behaviors, indicating that BMI may modify the association between eye rubbing and KC. However, no significant additive interactions were identified, suggesting that evidence for synergistic biological effects between these factors is limited. Lower education levels and childhood residence in remote areas were also linked to greater KC severity, likely resulting from reduced health literacy and limited access to care [27,28]. The frequency and intensity of eye rubbing were also major contributors to the development of KC, with intensity being particularly correlated with disease severity [29]. Additionally, AC was identified as an independent risk factor, without interaction with eye rubbing, likely owing to its inflammatory effects on the cornea and conjunctiva [6].

Note that a nonsignificant result in univariable logistic regression does not eliminate a factor as a potential disease risk, as its significance may be masked by other variables. In multivariable regression, several factors initially deemed significant in univariable logistic regression were excluded, likely because they acted as intermediary or confounding variables strongly correlated with other factors (e.g., childhood family socioeconomic status and childhood residence) [30]. This highlights a limitation of statistical logistic regression in capturing complex relationships and interactions when identifying both potential and independent risk factors [31].

To further explore KC risk factors and enhance the interpretability and simplicity of the ML model, we used univariable logistic regression, along with RF-RFE and XGBoost-RFE, for feature selection. Ultimately, we took the union of the features identified by XGBoost-RFE and those from univariable logistic regression, supplementing the potential risk factors highlighted through univariable analysis. The significant overlap between the intersection of the three methods and the multivariable analysis underscores the stability and reproducibility of the identified independent risk factors. By integrating the strengths of multiple methods and mitigating their limitations, ensemble approaches provide greater accuracy and stability than relying on a single feature-selection technique in ML [32].

We used seven ML algorithms to develop risk-classification models. LR was selected as a benchmark linear model because of its simplicity, maturity, and widespread use [33]. Tree-based methods, particularly RF and SVM, are highly effective for binary classification tasks and are commonly employed in the development of clinical prediction models [34]. Additionally, gradient-boosted decision trees (GBDTs), such as XGBoost, LightGBM, and CatBoost, were included because of their outstanding performance on tabular data [35] and demonstrated improvements in predictive accuracy for healthcare applications [36]. Furthermore, we incorporated NNs, which are particularly adept at capturing complex nonlinear relationships and often outperform traditional ML methods [33].

Among the models assessed, the NN demonstrated the best discrimination and calibration, achieving an AUROC value of 0.790, which signifies improved performance compared with traditional logistic regression (AUROC: 0.763). Notably, the AUROC of statistical logistic regression may be inflated owing to the lack of distinction between the training and test sets during calculation. The performance improvement can be attributed to the ML model’s ability to detect subtle patterns in complex data [37]. Despite this improvement, our NN model’s performance is still considered moderate based on its AUROC. It is essential to recognize that perfect classification is inherently unattainable, as KC results from a complex interplay of genetic and environmental factors, and not all individuals with high-risk factors will develop the condition [38]. In the ML algorithm comparison, the NN performed slightly better than tree-based models on our dataset, which is relatively small and has a moderate number of features. This finding is consistent with those of previous studies suggesting that the relative performance of NNs and GBDTs depends on data characteristics [39]. However, given the lack of significant differences, these results should be interpreted as comparable performance across models rather than the clear superiority of any single method.

Explainable ML allows us to evaluate models beyond mere performance metrics. Models using the same set of features may interpret their significance differently while achieving similar performance, as shown by the SHAP analysis in this study. This variation arises from the distinct mechanisms of different algorithms. For example, tree-based models such as RF and XGBoost select features at each split based on information gain or impurity reduction. RF, an ensemble method, builds independent decision trees and averages their outcomes, leading to a more distributed feature importance ranking. In contrast, XGBoost, a boosting algorithm, constructs each new tree to correct errors from previous iterations, assigning greater importance to features that contribute more significantly to the model’s improvement. Tree-based models generally prioritize features with high variability and discriminative power.

NNs, in contrast, learn complex relationships by adjusting the weights between neurons, allowing them to identify features that might be insignificant in isolation but crucial in combination with others. This explains the progressively decreasing overlap in the top 10 features identified by RF, XGBoost, and NN compared with those identified by univariable logistic regression. Despite differences in feature rankings across models, the consistent identification of key features—eye-rubbing intensity, sex, BMI, and childhood residence—across various methods, including multivariable logistic regression and feature selection, reinforces confidence in the robustness of the findings.

No definitive approach exists for determining feature importance, whether through traditional statistical methods or ML algorithms, making it difficult to assess which model’s ranking is more reliable. Still, understanding these rankings is crucial for building trust in healthcare applications, ensuring that clinicians and patients can confidently rely on the decisions generated by these models [40].

The analysis of KC risk factors in this study presents notable advantages compared with prior research. By using comprehensive datasets, we developed an evaluation system that goes beyond isolated risk factors, considering the intricate interplay among factors for personalized patient assessment. Notably, unlike imaging-based models that rely on specialized ophthalmic examinations, our approach uses lifestyle- and questionnaire-derived variables, providing a more accessible and non-invasive tool for early risk stratification at the population level. Although the predictive performance of such data alone is moderate, this framework establishes a foundation for future integrative models that combine lifestyle factors with objective corneal measurements. Further, automatic feedback based on electronic health records is facilitated by integrating ML [41]. In essence, our endeavor provides the groundwork for a more personalized approach, fostering data-driven individualized medicine.

## Limitations

This study has several limitations. First, the use of questionnaire-based data may have introduced recall bias, reporting bias, and potential reverse causation. Some factors, such as eye rubbing intensity, were self-reported and inherently subjective, potentially introducing differential reporting and measurement variability; furthermore, these factors may be more likely to be reported after diagnosis. Second, the control group consisted of myopic individuals seeking refractive surgery, which may have introduced selection bias. Myopia and related demographic factors may act as potential confounders and influence the observed associations with KC. In addition, some individuals in the control group may have had early KC that was not detectable at enrollment, leading to possible misclassification and overlap between groups. Third, the retrospective and cross-sectional design of this study precludes the establishment of temporal relationships, and the identified factors should be interpreted as associations rather than as causal relationships. Finally, as the data were derived from a single tertiary center, the generalizability of the findings may be limited. Multicenter studies and external validation are needed to confirm the robustness and broader applicability of the results. The model developed in this study is a lifestyle-based classification tool for individuals with KC at enrollment rather than a predictive model, and its implications for prevention require further validation in prospective longitudinal studies.

## Supporting information

S1 File Ordinal logistic regression and interaction effects among risk factors. (DOCX)

S1 Table Ordinal logistic regression model estimation results. (DOCX)

S2 Table Multiplicative interaction between eye rubbing and allergic conjunctivitis on keratoconus risk. (DOCX)

S3 Table Multiplicative interaction between eye rubbing and BMI on keratoconus risk. (DOCX)

S4 Table Detailed list of variables included in machine learning models. (DOCX)

S5 Table De-identified dataset of study participants used for analysis. (XLSX)
