# Supplementary material for: Identification and risk-factor analysis for individuals at high risk for keratoconus via machine learning and logistic regression
Source: PLoS One. 2026 Aug 25;21(8):e0354924. doi: 10.1371/journal.pone.0354924 (PMC13505905; doi:10.1371/journal.pone.0354924)
Supplement: Supplementary file 1 [file pone.0354924.s001.docx]

## Ordinal Logistic Regression

The severity variable was classified into four ordered levels, namely, “control,” “mild,” “moderate,” and “severe,” in order to explore the factors influencing KC severity. The relative impact of risk factors on severe compared to minor KC are shown in S1 Table. Ordered multinomial logistic regression analysis revealed that male sex, higher BMI, lower education level, more distant childhood residence, and increased eye rubbing intensity are associated with increased disease severity.

## Interactive Effects

We investigated the interaction between eye rubbing and AC history and found no statistically significant interactions on either the multiplicative or additive scale. Six interaction groups and three reference groups were defined based on eye-rubbing frequency, duration, and intensity among individuals without AC. These variables were incorporated into a stepwise multivariable logistic regression model together with other potential risk factors. In the final model, only the interaction term “rarely or never rubbing eyes without AC” was retained; however, it did not reach statistical significance (OR, 9.21; 95% CI, 0.92–92.02; *P* = 0.06), indicating no evidence of a multiplicative interaction between eye rubbing and AC history (S2 Table). Consistently, additive interaction analysis using the relative excess risk due to interaction also showed no significant interaction effects (all *P* > 0.05).

We further evaluated the interaction between eye rubbing and BMI. Interaction terms incorporating rubbing frequency, duration, and intensity across BMI categories were analyzed. Stepwise regression identified several significant multiplicative interactions, including “BMI 18.5–23.9 with occasional eye rubbing” (OR, 0.41; 95% CI, 0.26–0.66; *P* < 0.001), “BMI 24.0–27.9 with occasional eye rubbing” (OR, 0.58; 95% CI, 0.36–0.94; *P* = 0.03), and “BMI < 18.5 with light rubbing intensity” (OR, 0.28; 95% CI, 0.09–0.86; *P* = 0.03), suggesting significant multiplicative interactions between BMI and eye-rubbing behaviors (S3 Table). On the additive scale, no significant interactions were identified (all *P* > 0.05).
