# Supplementary material for: Identification and risk-factor analysis for individuals at high risk for keratoconus via machine learning and logistic regression
Source: PLoS One. 2026 Aug 25;21(8):e0354924. doi: 10.1371/journal.pone.0354924 (PMC13505905; doi:10.1371/journal.pone.0354924)
Supplement: Supplementary file 2 [file pone.0354924.s002.docx]

**S1 Table. Ordinal logistic regression model estimation results.**

| Categories | Characteristics | b | SE (b) | *P* value^a^ | OR (95% CI) |
| --- | --- | --- | --- | --- | --- |
| Sex | Male | 0.70 | 0.19 | < 0.001 | 2.01 (1.40–2.89) |
|  | Female | Reference | | | |
| BMI, | < 18.5 | –1.06 | 0.41 | 0.01 | 0.35 (0.15–0.78) |
| kg/m^2^ | 18.5–23.9 | –0.85 | 0.29 | 0.003 | 0.43 (0.24–0.76) |
|  | 24.0–27.9 | –0.49 | 0.31 | 0.11 | 0.61 (0.34–1.11) |
|  | ≥ 28 | Reference | | | |
| Education level | High school and below | 0.66 | 0.29 | 0.02 | 1.94 (1.09–3.46) |
|  | Undergraduate | –0.13 | 0.19 | 0.50 | 0.88 (0.60–1.28) |
|  | Master’s degree and above | Reference | | | |
| Childhood residence | Rural | 0.80 | 0.26 | 0.002 | 2.22 (1.34–3.66) |
|  | Small city | 0.32 | 0.26 | 0.22 | 1.38 (0.82–2.30) |
|  | Medium city | 0.09 | 0.30 | 0.78 | 1.09 (0.60–1.98) |
|  | Large city | –0.29 | 0.33 | 0.38 | 0.75 (0.40–1.42) |
|  | Mega city | Reference | | | |
| Eye rubbing frequency | Never/Rarely | –0.10 | 0.30 | 0.74 | 0.91 (0.50–1.63) |
|  | Occasionally | –0.43 | 0.27 | 0.11 | 0.65 (0.38–1.10) |
|  | Sometimes | –0.24 | 0.26 | 0.35 | 0.79 (0.47–1.30) |
|  | Frequently/Always | Reference | | | |
| Eye rubbing intensity | Light | –1.39 | 0.33 | < 0.001 | 0.25 (0.13–0.48) |
|  | Medium | –0.61 | 0.31 | 0.05 | 0.54 (0.30–0.99) |
|  | Heavy | Reference | | | |
| Allergic conjunctivitis | Yes | 0.53 | 0.33 | 0.11 | 1.70 (0.89–3.26) |
|  | No | Reference | | | |

Abbreviations: BMI, body mass index; CI, confidence interval; OR, odds ratio; SE, standard error.

^a^Comparisons were made by ordinal logistic regression.
