# Supplementary material for: Identification and risk-factor analysis for individuals at high risk for keratoconus via machine learning and logistic regression
Source: PLoS One. 2026 Aug 25;21(8):e0354924. doi: 10.1371/journal.pone.0354924 (PMC13505905; doi:10.1371/journal.pone.0354924)
Supplement: Supplementary file 3 [file pone.0354924.s003.docx]

**S2 Table. Multiplicative interaction between eye rubbing and allergic conjunctivitis on keratoconus risk.**

| **Categories** | **Characteristics** | **Interaction term** | ***P* value^a^** | **OR (95% CI)** |
| --- | --- | --- | --- | --- |
| **Eye rubbing frequency** | Never/Rarely | Retained | 0.06 | 9.21 (0.92–92.02) |
|  | Occasionally | Excluded |  |  |
|  | Sometimes | Excluded |  |  |
|  | Frequently/Always | Reference |  |  |
| **Eye rubbing duration** | < 10 seconds | Excluded |  |  |
|  | ≥ 10 seconds | Reference |  |  |
| **Eye rubbing intensity** | Light | Excluded |  |  |
|  | Medium | Excluded |  |  |
|  | Heavy | Reference |  |  |

Abbreviations: CI, confidence interval; OR, odds ratio.

^a^ Comparisons were made by stepwise logistic regression.
