# Supplementary material for: Identification and risk-factor analysis for individuals at high risk for keratoconus via machine learning and logistic regression
Source: PLoS One. 2026 Aug 25;21(8):e0354924. doi: 10.1371/journal.pone.0354924 (PMC13505905; doi:10.1371/journal.pone.0354924)
Supplement: Supplementary file 4 [file pone.0354924.s004.docx]

**S3 Table. Multiplicative interaction between eye rubbing and BMI on keratoconus risk.**

| **Categories** | **Characteristics** | **BMI** | **Interaction term** | ***P* value^a^** | **OR (95% CI)** |
| --- | --- | --- | --- | --- | --- |
| **Eye rubbing frequency** | Never/Rarely | < 18.5 | Excluded |  |  |
|  |  | 18.5–23.9 | Excluded |  |  |
|  |  | 24.0–27.9 | Excluded |  |  |
|  |  | ≥ 28 | Reference |  |  |
|  | Occasionally | < 18.5 | Excluded |  |  |
|  |  | 18.5–23.9 | Retained | < 0.001 | 0.41 (0.26–0.66) |
|  |  | 24.0–27.9 | Retained | 0.03 | 0.58 (0.36–0.94) |
|  |  | ≥ 28 | Reference |  |  |
|  | Sometimes | < 18.5 | Excluded |  |  |
|  |  | 18.5–23.9 | Excluded |  |  |
|  |  | 24.0–27.9 | Excluded |  |  |
|  |  | ≥ 28 | Reference |  |  |
|  | Frequently/Always |  | Reference |  |  |
| **Eye rubbing duration** | < 10 seconds | < 18.5 | Excluded |  |  |
|  |  | 18.5–23.9 | Excluded |  |  |
|  |  | 24.0–27.9 | Excluded |  |  |
|  |  | ≥ 28 | Reference |  |  |
|  | ≥ 10 seconds |  | Reference |  |  |
| **Eye rubbing intensity** | Light | < 18.5 | Retained | 0.03 | 0.28 (0.09–0.86) |
|  |  | 18.5–23.9 | Excluded |  |  |
|  |  | 24.0–27.9 | Excluded |  |  |
|  |  | 28 | Reference |  |  |
|  | Medium | < 18.5 | Excluded |  |  |
|  |  | 18.5–23.9 | Excluded |  |  |
|  |  | 24.0–27.9 | Excluded |  |  |
|  |  | ≥ 28 | Reference |  |  |
|  | Heavy |  | Reference |  |  |

Abbreviations: BMI, body mass index; CI, confidence interval; OR, odds ratio.

^a^ Comparisons were made by stepwise logistic regression.
