# Supplementary material for: Identification and risk-factor analysis for individuals at high risk for keratoconus via machine learning and logistic regression
Source: PLoS One. 2026 Aug 25;21(8):e0354924. doi: 10.1371/journal.pone.0354924 (PMC13505905; doi:10.1371/journal.pone.0354924)
Supplement: Supplementary file 5 [file pone.0354924.s005.docx]

**S4 Table. Detailed list of variables included in machine learning models.**

| **Variables** | **Variable type** | **Preprocessing method** | **Description** |
| --- | --- | --- | --- |
| Sex | Binary | Ordinal encoding | Male (0), Female (1) |
| Age, years | Categorical | Ordinal encoding | < 18 (0), 18-35 (1), > 35 (2) |
| BMI, kg/m^2^ | Continuous | Standardization |  |
| Education level | Categorical | Ordinal encoding | High school and below (0), Undergraduate (1), Master’s degree and above (2) |
| Occupational Status | Categorical | One-hot encoding | Office work (0), Non-office work (1), Unemployed/Retired (2), Student (3) |
| Childhood residence | Categorical | Ordinal encoding | Rural (0), Small city < 200,000 population (1), Medium city 200,000–500,000 (2), Large city 500,000–1,000,000 (3), Mega city > 1,000,000 (4) |
| Childhood family socioeconomic status | Categorical | Ordinal encoding | Poor (0), Average (1), Good/Excellent (2) |
| Eye rubbing frequency | Categorical | Ordinal encoding | Never/Rarely (0), Occasionally (1), Sometimes (2), Frequently/Always (3) |
| Eye rubbing duration | Binary | Ordinal encoding | < 10 seconds (0), ≥ 10 seconds (1) |
| Eye rubbing intensity | Categorical | Ordinal encoding | Light (0), Medium (1), Heavy (2) |
| Eye rubbing position | Categorical | Ordinal encoding | Fingertip (0), Knuckle (1), Whole fingertip (2), Palm root/Whole fist (3) |
| Rubbed eye area | Categorical | One-hot encoding | Eye corner (0), Eyelid (1), No difference (2) |
| Sleeping position | Categorical | Ordinal encoding | Back position (0), Side position (1), Prone position (2) |
| Use of eye coverings during sleep | Binary | Ordinal encoding | Yes (0), No (1) |
| Snoring and sleep apnea | Binary | Ordinal encoding | Yes (0), No (1) |
| Daily electronic screen time, hours | Categorical | Ordinal encoding | 0 (0), 1-3 (1), 4-6 (2), 7-9 (3), ≥ 10 (4) |
| Dark environment screen time, hours | Categorical | Ordinal encoding | 0 (0), 1-2 (1), 3-4 (2), 5-6 (3), ≥ 7 (4) |
| Daily paper material reading time, hours | Categorical | Ordinal encoding | 0 (0), 1-3 (1), 4-6 (2), 7-9 (3), ≥ 10 (4) |
| Daily sunlight/UV exposure time, hours | Categorical | Ordinal encoding | 0 (0), 1-3 (1), 4-6 (2), 7-9 (3), ≥ 10 (4) |
| Work/Study stress | Binary | Ordinal encoding | Yes (0), No (1) |
| Nighttime work/study | Binary | Ordinal encoding | Yes (0), No (1) |
| Allergic conjunctivitis | Binary | Ordinal encoding | Yes (0), No (1) |
| Allergic diseases | Binary | Ordinal encoding | Yes (0), No (1) |
| Alcohol consumption | Binary | Ordinal encoding | Yes (0), No (1) |

Abbreviations: BMI, body mass index; UV, ultraviolet.
